# Changes in human immunodeficiency virus and sexually transmitted infections-related sexual risk taking among young Croatian adults: findings from the 2005 and 2010 population-based surveys

**DOI:** 10.3325/cmj.2011.52.458

**Published:** 2011-08

**Authors:** Ivan Landripet, Aleksandar Štulhofer, Valerio Baćak

**Affiliations:** Sexology Unit, Department of Sociology, Faculty of Humanities and Social Sciences, University of Zagreb, Zagreb, Croatia

## Abstract

**Aim:**

To determine changes in sexual behaviors and other relevant characteristics related to human immunodeficiency virus (HIV) and sexually transmitted infection (STI) risks among young Croatian adults.

**Method:**

We surveyed adults aged 18-24 in 2005 (n = 1092) and 18-25 in 2010 (n = 1005). Both samples were probabilistic and stratified by county, settlement size, age, and gender. The samples were non-matched. Trained interviewers conducted structured face-to-face interviews in participants’ households. The part of the questionnaire assessing sensitive information was self-administered.

**Results:**

A majority of participants at both survey points (85.2%-86.2%) were sexually active. Median age at sexual debut (17 years) remained unchanged. Lifetime number of sexual partners was also stable. More women than men reported only one lifetime sexual partner. The prevalence of condom use at first intercourse increased (from 62.6 to 70%, *P* = 0.002), while the prevalence of condom use at most recent sexual intercourse remained stable (54% in 2005 and 54.7% in 2010). Consistent condom use also remained unchanged. About one fifth of participants (19.2% in 2005 and 20% in 2010) reported consistent condom use in the past year. At both survey points for both genders, consistent condom use was associated with age (odds ratio [OR] _Women2005_ = 0.74, *P* = 0.004; OR_Women2010_ = 0.72, *P* < 0.001; OR_Men2005_ = 0.73, *P* < 0.001; OR_Men2010_ = 0.80, *P* = 0.006), negative attitudes toward condom use (OR_Women2005_ = 0.84, *P* = 0.001; OR_Women2010_ = 0.90, *P* = 0.026; OR_Men2005_ = 0.92, *P* = 0.032; OR_Men2010_ = 0.90, *P* = 0.011)), and condom use at first intercourse (OR_Women2005_ = 3.87, *P* < 0.001; OR_Women2010_ = 4.64, *P* < 0.001; OR_Men2005_ = 5.85, *P* < 0.001; OR_Men2010_ = 4.03, *P* < 0.001). In the observed period, HIV/AIDS knowledge was stable.

**Conclusion:**

Risky sexual practices remain common among young Croatian adults. Given the recently reported STI prevalence rates in this age cohort, introduction of school-based sex education that would focus on protective behavioral and communication skills seems to be of crucial epidemiological importance.

Adolescents and young adults are more vulnerable to sexually transmitted infections (STI) than other segments of the general population. Inclined to intense exploration of their sexuality (1), young people are more likely to frequently change sexual partners, have multiple, often high-risk partners, and experiment with different sexual practices (2). In the same time, they often lack comprehensive knowledge of risks related to sexual health, as well as the communication and behavioral skills required for safer sex (3). It is therefore no surprise that most studies on youth sexuality focus on negative consequences of sexual activities (4-8).

A recent acquired immunodeficiency syndrome (AIDS) epidemic update suggested that almost half of all new human immunodeficiency virus (HIV) infections worldwide occurred among people aged 15-24 years (9). One study of adolescents in the United States of America found that about 50% of all newly contracted STIs were reported among adolescents and young adults, with human papillomaviruses (HPV), trichomoniasis, and *Chlamydia trachomatis* being the most frequently acquired STIs (10). Although we lack biological data on STIs in Croatia, the existing data on HPV suggest that the prevalence of STIs in the country might be comparable. As recently observed, vulnerability to HPV infections seems to be highest among women in their late teens and early twenties (11,12). This is not surprising given the well-documented inconsistency of condom use in the population (3,13-17). The situation is not substantially better among well-educated young adults. In a cross-sectional study carried out in 1998, 2003, and 2008 among the University of Zagreb first-year students, fewer than a half of participants reported using condoms regularly (18).

Unfortunately, there is no comprehensive sex education in Croatian public schools. The recent debates about its introduction proved highly controversial and politicized (19). As a result, the initiative was officially qualified as an unnecessary burden to the national curriculum and dropped (20). Under such circumstances, systematic monitoring of sexual risks among young people is an important public health task. The aim of this article is to provide evidence-based rationale for interventions and educational programs focusing on reproductive and sexual health issues. In this first repeated cross-sectional study based on national probability samples carried out in Croatia, we examine core indicators of sexual risk taking and other relevant characteristics of young adults in the period 2005-2010 to inform a national response to HIV and STI risks among young people.

## Method

### Sampling procedure

In 2005 and 2010, two cross-sectional studies on HIV/AIDS-related attitudes, knowledge, and behaviors were carried out on national probability non-matched samples of young adults. Using data from the last census (2001), both samples were stratified according to county, settlement size, age, and gender. Consistent randomization procedures were employed in all sampling steps. In the last step, Kish’s method of the most recent birthday (21) was used to select participants in the households where more than one resident of the eligible age was present.

In 2005, the number of participants was set equal for all 6 regions of the country to enable robust regional estimates (13), while in 2010 probability proportional to size sampling approach was used. To make comparisons between the two waves meaningful, the 2005 data set was weighted to adjust for regional differences, settlement size, age, and gender. At 95% confidence interval, the maximum margin of error for the 2005 sample was ±2.97%, and for the 2010 sample ±3.09%.

### Participants

The 2005 survey included 1092 women and men aged 18-24 years, while the 2010 survey included 1005 participants aged 18-25 years. This slight difference in age ranges may seem problematic for between-study comparisons, however a set of analyses suggested that this was not the case. The inclusion of 25-year-olds in 2010 did not affect comparisons of age at coital debut. All sexually active participants in the 2010 sample reported having had first sexual intercourse at the age of 24 years or younger. Furthermore, 25-year-olds did not significantly differ from 24-year-olds in the lifetime number of sexual partners (χ^2^ = 3.07, *P* = 0.381), the reported number of partners in the past year (χ^2^ = 7.72, *P* = 0.102), condom use at most recent intercourse (χ^2^ = 5.49, *P* = 0.241), and consistent condom use in the past month (χ^2^ = 0.12, *P* = 0.911).

### Non-response analysis

In 2005, the survey had a high response rate of 79.5%. Five years later, as many as 3133 persons of eligible age and gender were approached and 1005 completed the questionnaire, yielding a response rate of 32.1%. Although considerably lower, this rate is comparable to some of the recently observed participation rates in Croatian public opinion surveys and corresponds to the long-term negative trend in survey participation (22,23).

The interviewers were instructed to substitute an eligible person that declined participation according to a standardized randomization procedure. Among those who refused to take part in the survey, 68.9% in 2005 and 65.4% in 2010 reported lack of time or a general disinterest in surveys. Additional 7.7% (2005) and 10.8% (2010) stated health issues, their parents’ disapproval, and other (or no) reasons. It is important to note that the structure of non-response was almost identical when refusal was attributed to the study topic (HIV-relevant knowledge, beliefs, and behaviors) – 23.4% in 2005 and 23.8% in 2010.

### Data collection

Participants were interviewed in their homes or, exceptionally, at some other nearby public place (park, coffee shop, etc.) where it was possible to conduct the interview without the presence of other family members. Most interviewers were young women between 25 and 35 years of age, with considerable interviewing experience. Both in 2005 and 2010, the interviewers received an additional six-hour training focused on collecting information on sensitive topics.

All study procedures were approved by the Ethical Review Board of the Faculty of Humanities and Social Sciences, University of Zagreb. Prior to the interview, all participants gave verbal informed consent. Following the completion of the survey, participants were given brochures with essential HIV/AIDS information. The 2010 survey additionally included a biological part (not reported here). After participants had completed the survey, they were asked for a urine sample to be tested for chlamydia. Prior to completing the questionnaire, participants were unaware of the biological testing component.

### Questionnaire

An originally developed KABP (knowledge, attitudes, beliefs, and practices) questionnaire was used in both waves, with slight modifications. The first part of the questionnaire, administered by face-to-face interviewing, asked about socio-demographic characteristics, HIV/AIDS knowledge, attitudes toward people living with HIV/AIDS, attitudes toward gendered sexual roles, beliefs about condoms and condom use, self-esteem, and locus of control. The second part of the questionnaire, focused on sexual behaviors and other relevant experiences (including the use of pornography, having been diagnosed with an STI, and having been tested for HIV), was self-administered. The questionnaires consisted of 170-190 items and took on average 30 minutes to complete. The 2005 version was piloted for comprehensiveness and completion time among 100 high-school students. Slightly revised version was pre-tested in 2009 on 103 high-school and 132 university students. Instruments used for comparisons were identical in the original and the repeated study.

### Measures

Socio-demographic characteristics assessed were age and personal and parental education (obtained separately for mother and father), occupation, family socio-economic status, and the type of the longest place of residence (1 = <10 000 inhabitants, 2 = 10 000-50,000, 3 = 50 001-100,000, 4 = 100 001-500,000, 5 = >500,000). In addition, two measures of religiosity were used. Religious upbringing was measured by the following single-item indicator: “Were you brought up in the religious spirit?” A three-point scale was used to anchor answers (1 = no, 2 = yes, but not strictly, and 3 = yes, strictly). Personal religiosity was assessed by the frequency of attending religious services on occasions other than weddings, funerals, christenings, and the like, ranging from 1 = never to 6 = almost daily.

Knowledge about HIV/AIDS was measured with the standard UNGASS indicators (24). Five items assessed routes of HIV transmission, while 2 additional items measured modes of protection from HIV infection (Table 1). Answers were recoded as correct and incorrect (including “don't know” answers). Items were summed to form a composite indicator ranging from 0 (all incorrect answers) to 7 (all correct answers).

**Table 1:** Knowledge about HIV/AIDS by study year and gender*

	No. (%) of participants in:					
	2005			2010		
	women	men	total	women	men	total
Can a person get HIV:						
from mosquito bites	361 (67.1)	339 (61.2)	700 (64.1)	348 (70.3)	312 (61.2)	660 (65.7)
using public toilets	398 (74.1)	418 (75.5)	816 (74.7)	376 (76.0)	387 (75.9)	763 (75.9)
sharing a glass with someone who is infected^†^	391 (72.7)	389 (70.2)	780 (71.4)	401 (81.0)	379 (74.3)	780 (77.6)
sharing a meal with someone who is infected	412 (76.6)	397 (71.7)	809 (74.1)	393 (79.4)	374 (72.3)	767 (76.3)
having sex with a healthy looking person^†^	466 (86.6)	470 (84.8)	936 (85.7)	446 (90.1)	462 (90.6)	908 (90.3)
Can the risk of HIV transmission be reduced by:						
proper use of condoms	453 (84.2)	455 (82.1)	908 (83.2)	375 (75.8)	428 (83.9)	803 (79.9)
having sex with only one faithful and uninfected partner‡	434 (80.7)	412 (74.4)	846 (77.5)	345 (69.7)	388 (76.1)	733 (72.9)

*Number (percentage) of correct answers.

†Significant difference between the study waves (totals), *P* < 0.01.

‡Significant difference between the study waves (totals), *P* < 0.05.

Attitudes toward condom use were assessed by a four-item Negative Beliefs about Condom Use Scale developed by A. Štulhofer and colleagues (13). Items such as “A person who suggests condom use does not trust his/her partner.” and “A girl who carries condoms in her purse can be easily talked into having sex.” were anchored on a 5-point Likert-type scale ranging from 1 = “completely disagree” to 5 = “completely agree.” An additive composite scale ranging from 4 to 20 (the higher result, the more negative beliefs about condom use) was one-dimensional in both waves (all items loaded >0.70 on one factor with Eigenvalue >1) and had acceptable internal consistency (Cronbach α_2005_ = 0.75; α_2010_ = 0.83).

Socio-sexual characteristics and sexual risk-taking behaviors were assessed with several single-item indicators: age at sexual debut (defined as first coital intercourse), contraception/protection use at first and most recent sexual intercourse, condom use consistency (in the past 12 months), and number of sexual partners (lifetime and during the last 12 months). Sexual partners were defined as individuals the participant had vaginal intercourse with. Sexual orientation was assessed by asking about the gender of sexual partners (from 1 = “exclusively men” to 5 = “exclusively women”). Self-assessed HIV and STI-related risks were measured by the following questions: “How would you rate your personal risk of acquiring HIV infection?” and “How would you rate your personal risk of acquiring any other STI?” A 10-point response scale ranging from 1 (“negligible risk”) to10 (“extremely high risk”) was used for anchoring answers. Finally, participants were asked to indicate whether they had ever been diagnosed with an STI and whether they had ever been tested for HIV.

### Statistical analysis

χ^2^ tests were used to assess differences between genders and study waves on various indicators. *t*-tests were used to compare study wave and gender-specific means on several continuous measures, which were analyzed for normality of distribution. Reliability analysis and principal component analysis were carried out to assess internal consistency and dimensionality of composite indicators. Consistent condom use, as the central indicator of responsible sexual behavior among young adults with multiple partnerships, was assessed in more detail with multivariate logistic regression analysis. The analyses were carried out separately by study wave and gender. The model included the variables found relevant in previous studies (7,13,14,17). All statistical analyses were performed with SPSS 17.0 (SPSS Inc., Chicago, IL, USA) statistical software package. Probability value <0.05 was set as a threshold for statistical significance.

## Results

Young adults sampled in 2005 and 2010 significantly differed in most socio-demographic characteristics (Table 2). Although the proportion of participants with strict religious upbringing significantly increased (χ^2^ = 99.98, *P* < 0.001), the frequency of church-going slightly but significantly decreased (χ^2^ = 13.92, *P* = 0.003).

**Table 2:** Socio-demographic structure of the samples by study year and gender

	No. (%) of participants in:					
	2005			2010		
	women (n = 574)	men (n = 519)	total (N = 1093)	women (n = 495)	men (n = 510)	total (N = 1005)
Father's education:*						
elementary school or less	80 (15.1)	51 (9.4)	131 (12.2)	45 (9.3)	41 (8.1)	86 (8.7)
high school	361 (68.1)	397 (73.1)	758 (70.6)	329 (67.7)	338 (66.7)	607 (67.2)
university degree	89 (16.8)	95 (17.5)	184 (17.2)	112 (23.0)	128 (25.2)	240 (24.2)
Mother's education:^†^						
elementary school or less	85 (15.9)	105 (19.0)	190 (17.5)	62 (12.5)	59 (11.6)	121 (12.1)
high school	348 (65.2)	333 (60.2)	681 (62.7)	334 (67.5)	348 (68.4)	682 (67.9)
university degree	101 (18.9)	115 (20.8)	216 (19.8)	99 (20.0)	102 (20.0)	201 (20.0)
Family socioeconomic status:*						
lower than average	49 (9.1)	42 (7.6)	91 (8.4)	16 (3.2)	21 (4.1)	37 (3.7)
about average	382 (71.3)	391 (71.1)	773 (71.2)	367 (74.1)	365 (71.6)	732 (72.8)
higher than average	105 (19.6)	117 (21.3)	222 (20.4)	112 (22.6)	124 (24.3)	236 (23.5)
Respondent’s occupation:						
in school/at university	288 (53.5)	264 (47.7)	552 (50.5)	273 (55.2)	229 (44.9)	502 (50.0)
employed	151 (28.1)	181 (32.7)	332 (30.4)	150 (30.3)	192 (37.6)	342 (34.0)
unemployed	99 (18.4)	109 (19.7)	208 (19.0)	72 (14.5)	89 (17.5)	161 (16.0)
Attendance of religious services:^†^						
never	130 (24.2)	174 (31.6)	304 (28.0)	147 (29.8)	189 (37.1)	336 (33.5)
up to several times a year	182 (33.9)	211 (38.4)	393 (36.2)	185 (37.4)	191 (37.5)	376 (37.5)
once a month	115 (21.4)	75 (13.6)	190 (17.5)	87 (17.6)	66 (12.9)	153 (15.2)
once a week or more	110 (20.5)	90 (16.4)	200 (18.3)	75 (15.2)	64 (12.5)	139 (13.8)
Raised religiously at home:*						
no	98 (18.2)	106 (19.4)	204 (18.9)	55 (11.1)	74 (14.6)	129 (12.9)
yes, but not strictly	371 (69.1)	396 (72.7)	767 (70.9)	299 (60.5)	302 (59.7)	601 (60.1)
strictly	68 (12.7)	43 (7.9)	111 (10.3)	140 (28.3)	130 (25.7)	270 (27.0)
No. of inhabitants in the settlement of longest residence:						
≤10,000	259 (48.9)	305 (55.2)	564 (52.1)	249 (50.4)	256 (50.5)	505 (50.4)
10,001-50,000	90 (17.0)	110 (19.9)	200 (18.5)	81 (16.4)	83 (16.7)	164 (16.4)
50,001-100,000	42 (7.9)	37 (6.7)	79 (7.3)	52 (10.5)	49 (9.7)	101 (10.1)
100,001-500,000	54 (10.2)	47 (8.5)	101 (9.3)	40 (8.1)	55 (10.8)	95 (9.5)
>500,000	85 (16.0)	54 (9.8)	139 (12.8)	72 (14.6)	64 (12.6)	136 (13.6)
Currently married^‡^	35 (6.5)	18 (3.2)	53 (4.9)	46 (9.3)	25 (4.9)	71 (7.1)

*Significant difference between the study waves (totals), *P* < 0.001.

†Significant difference between the study waves (totals), *P* < 0.01.

‡Significant difference between the study waves (totals), *P* < 0.05.

### HIV/AIDS knowledge

Basic HIV/AIDS knowledge was relatively high in both study waves (Table 1). With the exception of a single item (mosquito bites), between 70% and 90% of participants provided correct answers regarding HIV transmission. Similar proportions (70%-85%) of correct answers were recorded on two questions assessing the knowledge about protection from HIV infection. Slight but significant increase was observed for two items measuring modes of HIV transmission. About 6% more participants answered that HIV cannot be transmitted by sharing a glass with an infected person (χ^2^ = 10.5, *P* < 0.001) and about 5% more knew that healthy looking partner may still be HIV positive (χ^2^ = 10.59, *P* < 0.001). The scores on the overall additive index, however, remained the same in the observed period.

### Sexual experiences and behaviors

About 85% of participants in 2010 were sexually active (Table 3). The proportion did not significantly change in the observed period. Differences between genders in sexual activity were found only in 2005, when more men reported sexual activity (χ^2^ = 5.22, *P* = 0.022). Between 2005 and 2010, the average age at sexual debut decreased significantly in statistical (mean ± standard deviation in 2005, 17.31 ± 1.71; in 2010, 17.14 ± 1.75; *t* = 1.99, df = 1734, *P* = 0.047) but not in practical terms. When tested using non-parametric procedures, the connection between age at first intercourse and the study wave was not confirmed (χ^2^ = 10.79, df = 6, *P* = 0.095) (Table 3). In 2005, men reported sexual debut at a mean ± standard deviation of 17.02 ± 1.62 years and women at 17.61 ± 1.74 years (*t* = 5.19, df = 865, *P* < 0.001). In 2010, men reported sexual debut at a mean ± standard deviation of 16.9 ± 1.86 years and women at 17.4 ± 1.59 years (*t* = 4.23, df = 850, *P* < 0.001). In both study waves, the median age at sexual debut was 17 years (interquartile range: 16-18) for both genders. It should also be noted that a proportion of participants who reported sexual debut before the age of 17 remained stable.

**Table 3:** Sexual experiences and behaviors by study year and gender*

	No. (%) of participants in:					
	2005			2010		
	women	men	total	women	men	total
Experience of sexual intercourse:						
yes	440 (82.7)	482 (87.6)	922 (85.2)	416 (84.4)	445 (87.9)	861 (86.2)
Age at the first intercourse (years):						
≤14	10 (2.4)	25 (5.4)	35 (4.0)	8 (1.9)	36 (8.2)	44 (5.2)
15	22 (5.2)	42 (9.1)	64 (7.2)	36 (8.8)	53 (12.0)	89 (10.4)
16	85 (20.0)	96 (20.9)	181 (20.5)	72 (17.5)	101 (22.9)	173 (20.3)
17	116 (27.3)	123 (26.7)	239 (27.0)	109 (26.5)	100 (22.7)	209 (24.5)
18	65 (15.3)	112 (24.3)	177 (20.0)	98 (23.8)	86 (19.5)	184 (21.6)
19	63 (14.8)	30 (6.5)	93 (10.5)	47 (11.4)	32 (7.3)	79 (9.3)
≥20	64 (15.0)	32 (7.1)	96 (10.8)	41 (10.1)	33 (7.4)	74 (8.7)
Number of sexual partners (ever):						
1	147 (35.5)	63 (14.7)	210 (24.9)	121 (29.7)	64 (15.5)	185 (22.5)
2	84 (20.3)	60 (14.0)	144 (17.1)	73 (17.9)	37 (8.9)	110 (13.4)
3	52 (12.6)	79 (18.5)	131 (15.6)	77 (18.9)	57 (13.8)	134 (16.3)
4-5	74 (17.9)	91 (21.3)	165 (19.6)	76 (18.6)	86 (20.8)	162 (19.7)
6-9	37 (8.9)	64 (15.0)	101 (12.0)	33 (8.1)	80 (19.3)	113 (13.7)
≥10	20 (4.8)	71 (16.5)	91 (10.8)	28 (6.8)	90 (21.7)	118 (14.4)
Number of sexual partners in the last 12 mo:^†^						
0	38 (8.9)	43 (9.4)	81 (9.2)	17 (4.2)	23 (5.3)	40 (4.8)
1	291 (68.8)	220 (47.9)	511 (57.9)	298 (72.9)	211 (49.0)	509 (60.7)
2	54 (12.8)	89 (19.4)	143 (16.2)	58 (14.1)	79 (18.4)	137 (16.3)
3-4	35 (8.3)	79 (17.2)	114 (12.9)	27 (6.6)	63 (14.7)	90 (10.7)
≥5	5 (1.2)	28 (6.1)	33 (3.8)	9 (2.2)	54 (12.6)	63 (7.5)
Most recent intercourse was with a steady partner:						
yes	355 (84.7)	320 (69.3)	675 (76.6)	355 (86.4)	291 (65.8)	646 (75.7)
no	64 (15.3)	142 (30.7)	206 (23.4)	56 (13.6)	151 (34.2)	207 (24.3)
Concurrent sexual relationship (ever):						
no	353 (83.5)	316 (68.8)	669 (75.9)	342 (82.8)	314 (71.0)	656 (76.7)
yes	70 (16.5)	143 (31.2)	213 (24.1)	71 (17.2)	128 (29.0)	199 (23.3)
Gender of sexual partners:						
exclusively opposite	391 (92.4)	430 (93.1)	821 (92.9)	384 (93.2)	419 (95.0)	803 (94.1)
mostly opposite	26 (6.1)	23 (5.0)	49 (5.4)	22 (5.3)	9 (2.0)	31 (3.6)
equally opposite and same	4 (1.0)	3 (0.6)	7 (0.8)	4 (1.0)	1 (0.2)	5 (0.6)
mostly or exclusively same	2 (0.5)	6 (1.3)	8 (0.9)	2 (0.5)	12 (2.7)	14 (1.8)
Ever diagnosed with an STI:^†^						
yes	67 (15.4)	14 (3.0)	81 (9.0)	21 (5.1)	18 (4.1)	39 (4.6)
Ever been tested for HIV:^‡^						
yes	20 (4.6)	33 (7.0)	53 (5.9)	31 (7.5)	48 (10.8)	79 (9.2)

*Only participants who reported sexual intercourse were included.

^†^Significant difference between the study waves (totals), *P* < 0.001.

^‡^Significant difference between the study waves (totals), *P* < 0.01.

The lifetime number of sexual partners did not change between the study waves. The median number was 3 (interquartile range: 2-5 in 2005, 2-6 in 2010). As for the number of partners in the last year, the observed difference (χ^2^ = 25.0, *P* < 0.001) was generated primarily by a lower proportion of participants who were sexually inactive in 2010 compared to 2005, as well as a higher proportion of participants who reported 5 or more sexual partners. In both waves, more women than men reported only one sexual partner – in the past 12 months (χ^2^_2005_ = 50.38, *P* < 0.001; χ^2^_2010_ = 65.05, *P* < 0.001) and lifetime (χ^2^_2005_ = 80.51, *P* < 0.001; χ^2^_2010_ = 85.03, *P* < 0.001).

In 2005 and 2010, about three quarters of participants (75.9%-76.7%) had the most recent sexual intercourse with a steady partner. About one quarter of participants reported having had concurrent partnerships at least once during their lifetime (23.3%-24.1%). Differences between genders were observed for both indicators. In 2005, twice as many men (χ^2^ = 29.32, *P* < 0.001) and in 2010 2.5 times as many men than women (χ^2^ = 48.88, *P* < 0.001) reported that they had had most recent intercourse with a casual partner. Similarly, about two times more men than women reported concurrent partnerships (χ^2^_2005_ = 25.64, *P* < 0.001; χ^2^_2010_ = 16.56, *P* < 0.001).

HIV testing almost doubled in the 2005-2010 period (from 5.9 to 9.2%; χ^2^ = 7.60, *P* = 0.006), but the proportions remained fairly low (Table 3). No differences between genders in HIV testing were found (Table 3).

### Patterns of and attitudes toward condom use

Condom use at first intercourse increased from 62.6% in 2005 to 70% in 2010 (χ^2^ = 16.7, *P* = 0.002; Table 4). Importantly, about one fifth of participants in both study waves reported that they and their partner used no method or technique of protection from STIs and unwanted pregnancy. When condom use at most recent intercourse was examined, no significant difference between the waves was found. There were more participants in both study waves who reported that they or their partner had used a condom at most recent intercourse than those who reported it for the first intercourse (Table 4).

**Table 4:** Patterns of condom use and HIV/STI risk self-assessment by study year and gender*

	No. (%) of participants in:					
	2005			2010		
	women	men	total	women	men	total
Protection at the first intercourse:^†^						
none	90 (21.3)	94 (20.3)	184 (20.8)	56 (13.7)	107 (24.7)	163 (19.4)
withdrawal	66 (15.6)	55 (11.9)	121 (13.7)	48 (11.7)	31 (7.2)	79 (9.4)
condom	253 (60.0)	302 (65.1)	555 (62.6)	297 (72.6)	292 (67.4)	589 (70.0)
pill	10 (2.4)	9 (1.9)	19 (2.1)	5 (1.2)	3 (0.7)	8 (1.0)
other	3 (0.7)	4 (0.9)	8 (0.8)	3 (0.7)	0 (0.0)	3 (0.4)
Protection at most recent intercourse:						
none	97 (23.0)	93 (20.0)	190 (21.5)	87 (21.2)	118 (27.0)	205 (24.2)
withdrawal	56 (13.3)	45 (9.7)	101 (11.4)	56 (13.6)	35 (8.0)	91 (10.7)
condom	194 (46.1)	290 (62.5)	484 (54.7)	208 (50.6)	250 (57.2)	458 (54.0)
pill	59 (14.0)	27 (5.8)	86 (9.7)	48 (11.7)	24 (5.5)	72 (8.5)
other	15 (3.5)	9 (1.9)	24 (2.7)	12 (2.9)	10 (2.3)	22 (2.6)
Frequency of condom use in the last 12 mo:						
didn't have sex	29 (6.9)	28 (6.1)	57 (6.5)	10 (2.4)	10 (2.3)	20 (2.3)
never	78 (18.6)	52 (11.4)	130 (14.9)	88 (21.2)	61 (13.8)	149 (17.4)
rarely	79 (18.9)	95 (20.8)	174 (19.9)	80 (19.3)	90 (20.4)	170 (19.9)
sometimes	78 (18.6)	67 (14.7)	145 (16.6)	62 (14.9)	79 (17.9)	141 (16.5)
often	84 (20.0)	110 (24.1)	194 (22.2)	100 (24.1)	112 (25.4)	212 (24.8)
always	71 (16.9)	104 (22.8)	175 (20.0)	75 (18.1)	89 (20.2)	164 (19.2)
HIV risk self-assessment:^‡^						
non-existent	302 (70.2)	318 (67.8)	620 (69.0)	327 (80.3)	311 (71.5)	638 (75.8)
low	71 (16.6)	101 (21.5)	172 (19.2)	48 (11.8)	68 (15.6)	116 (13.8)
moderate	40 (9.2)	39 (8.2)	78 (8.7)	24 (5.9)	39 (9.0)	63 (7.5)
considerable	17 (4.0)	12 (2.5)	29 (3.1)	8 (1.9)	17 (3.9)	25 (3.0)
STI risk self-assessment:						
non-existent	276 (64.3)	276 (58.8)	552 (61.4)	248 (60.0)	262 (59.4)	510 (59.7)
low	73 (17.0)	121 (25.7)	194 (21.5)	95 (23.0)	262 (22.4)	194 (22.7)
moderate	60 (13.9)	47 (10.1)	107 (11.9)	55 (12.9)	57 (12.9)	112 (13.1)
considerable	21 (4.8)	26 (5.5)	46 (5.2)	15 (5.0)	23 (5.2)	38 (4.5)

*Only participants who reported sexual intercourse were included.

†Significant difference between the study waves (totals), *P* < 0.01.

‡Significant difference between the study waves (totals), *P* < 0.05.

Consistent use of condoms in the past 12 months did not increase between 2005 and 2010 (participants who were not sexually active during the past year were excluded from the analysis). About one fifth of participants reported using condoms regularly (19.2%-20%). These rates need also to be considered from the point of view of condom use failures and errors. In 2010 (the 2005 wave did not assess problems with condom use), as many as 34.1% of participants reported delayed condom application at least once in the past year and 18.1% condom breakage. Men were more likely to report condom breakage (χ^2^ = 6.27, *P* = 0.033).

Negative beliefs about condom use remained low. Compared to women, men scored higher on the scale, showing less positive attitudes and beliefs toward condoms in both the 2005 (mean ± standard deviation = 9.42 ± 3.57 in men and in women = 8.5 ± 3.36; *t* = 4.35, df = 1075, *P* < 0.001) and 2010 study (mean ± standard deviation = 9.89 ± 3.96 in men and in women = 8.32 ± 3.64; *t* = 6.51, df = 997, *P* < 0.001).

Regardless of the observed risks, a great majority of sexually active participants assessed the risk of becoming infected with HIV or other STIs as non-existent or marginal. In the case of HIV risks, the proportion of young adults holding this view even increased in the observed period (from 69% in 2005 to 75.8% in 2010; χ^2^ = 11.12, *P* = 0.011). In 2005, more women than men judged the risk of becoming infected with STIs as negligible (*χ^2^* = 12.23, *P* = 0.007). In 2010, the same tendency was observed with HIV risk self-assessment, with 80.3% of women and 71.5% of men judging their risk of HIV infection as negligible (χ^2^ = 9.74, *P* = 0.021) (Table 4).

### Correlates of consistent condom use

Consistent condom use was associated with age, condom use at first sexual intercourse, and attitudes toward condom use (Table 5). In both study waves, participants’ older age (odds ratio [OR]_women2005_ = 0.74, *P* = 0.004; OR_women2010_ = 0.72, *P* < 0.001; OR_men2005_ = 0.73, *P* < 0.001; OR_men2010_ = 0.80, *P* = 0.006) and negative attitudes toward condom use (OR_women2005_ = 0.84, *P* = 0.002; OR_women2010_ = 0.90, *P* = 0.026; OR_men2005_ = 0.92, *P* = 0.032; OR_men2010_ = 0.90, *P* = 0.011) were associated with lower odds of consistent condom use. Condom use at first sexual intercourse was by far the strongest predictor. Among women, it increased the odds of consistent condom use 3.84-4.64 times (*P* < 0.001). Among men, an association of similar magnitude was found (OR = 4.03-5.85; *P* < 0.001).

**Table 5:** Correlates of consistent condom use by study wave and gender*

	2005		2010	
	women	men	women	men
	OR (95% CI)^†^	OR (95% CI)	OR (95% CI)	OR (95% CI)
Age	0.74 (0.61-0.91)^‡^	0.73 (0.62-0.86)^§^	0.72 (0.61-0.85)^§^	0.80 (0.68-0.94)^‡^
Father’s education:				
elementary school or less	0.97 (0.25-3.80)	1.38 (0.39-4.87)	0.73 (0.07-7.36)	1.38 (0.35-5.43)
high school	0.56 (0.24-1.34)	0.53 (0.24-1.15)	1.57 (0.71-3.51)	1.00 (0.49-2.04)
university degree (referent)	1	1	1	1
Mother’s education:				
elementary school or less	1.10 (0.27-4.50)	1.09 (0.38-3.15)	0.15^||^ (0.03-0.72)	0.70 (0.20-2.47)
high school	1.97 (0.79-4.90)	1.16 (0.55-2.44)	0.52 (0.23-1.16)	0.54 (0.26-1.12)
university degree (referent)	1	1	1	1
Family socioeconomic status:				
average or lower (referent)	1	1	1	1
higher than average	0.56 (0.21-1.49)	0.72 (0.34-1.49)	1.28 (0.61-2.70)	1.72 (0.86-3.44)
Church attendance:				
never (referent)	1	1	1	1
up to several times a year	0.93 (0.39-2.21)	1.62 (0.84-3.13)	1.26 (0.60-2.64)	1.38 (0.71-2.65)
once a month	1.00 (0.36-2.60)	1.60 (0.67-3.80)	1.54 (0.62-3.81)	0.38 (0.12-1.25)
once a week or more	1.90 (0.72-5.02)	0.99 (0.41-2.40)	1.41 (0.51-3.89)	1.61 (0.57-4.54)
age at first sexual intercourse	1.46 (1.11-1.81)^‡^	1.23 (0.99-1.54)	1.19 (0.92-1.53)	1.13 (0.93-1.39)
Condom used at first sexual intercourse	3.87 (1.80-8.32)^§^	5.85 (2.79-12.24)^§^	4.64 (1.83-11.80)^§^	4.03 (1.76-9.22)^§^
Lifetime number of partners:				
1 (referent)	1	1	1	1
2-3	1.19 (0.56-2.52)	1.18 (0.51-2.73)	1.44 (0.70-2.98)	0.36 (0.14-0.89)^||^
4-5	1.91 (0.71-5.14)	1.31 (0.50-3.43)	0.48 (0.17-1.35)	0.29 (0.10-0.82)^||^
>6	1.94 (0.63-6.00)	1.22 (0.47-3.16)	0.68 (0.18-2.50)	0.36 (0.13-1.00)
HIV/AIDS knowledge:				
median score or lower (referent)	1	1	1	1
higher than median score	1.08 (0.57-2.07)	0.68 (0.37-1.24)	1.41 (0.75-2.64)	0.85 (0.44-1.67)
negative attitudes toward condom use	0.84 (0.75-0.93)^‡^	0.92 (0.84-0.99)^||^	0.90 (0.82-0.99)^||^	0.90 (0.83-0.98)^||^
Self-assessed HIV/STI risks	0.96 (0.88-1.06)	0.93 (0.85-1.01)	0.92 (0.81-1.04)	0.95 (0.86-1.06)
Tested for HIV	0.24 (0.02-2.46)	0.72 (0.22-2.33)	1.20 (0.39-3.73)	1.27 (0.46-3.55)

*Only participants who reported sexual intercourse were included.

†OR – odds ratio; CI – confidence interval.

‡Significant difference between the study waves (totals), *P* < 0.01.

§Significant difference between the study waves (totals), *P* < 0.001.

||Significant difference between the study waves (totals), *P* < 0.05.

A few other significant correlates were gender-specific and lacked temporal robustness. Higher odds of consistent condom use among women in 2005 were associated with older age at sexual debut (OR = 1.46, *P* = 0.006). In 2010, consistent condom use in women was associated with mother’s education – women who reported that their mothers had only elementary education were 85% less likely to consistently use condoms than women with college-educated mothers (OR = 0.15, *P* = 0.018). Among men, lower odds of consistent condom use in 2010 were associated with having more lifetime sexual partners (OR = 0.29-0.36, *P* = 0.02) (Table 5).

## Discussion

The primary aim of this study was to examine the change in HIV and STI-related sexual risk taking in the population of young Croatian adults during the 2005-2010 period. Our results corroborate the findings from earlier studies among Croatian adolescents and late adolescents (3,7,13,14,16-18). Although there is evidence that among young people condom use increased (25), a substantial proportion still engage in risky sexual practices, including having multiple and concurrent sexual partners and not using condoms consistently. The available data clearly indicate that young men and women in Croatia are exposed to an increased vulnerability to STIs.

Contrary to the often repeated fear of hyper-sexualization of youth in Croatia, the median age at sexual debut remained stable since the late 1980s (15,26). A recent cross-cultural study of 59 countries world-wide confirmed the absence of a universal decrease in age at first sexual intercourse (27). Our findings seem to corroborate this, as the 2005-2010 difference in the average age at sexual debut was very small (and likely an artifact of sample size). Likewise, lifetime number of sexual partners and the proportion of young men and women who reported concurrent sexual partnerships remained unchanged and comparable to a number of European countries (28-30).

From epidemiological perspective, however, the fact that a majority of young adults have multiple sexual partners remains of primary importance – regardless of whether the number is increasing or not. In the absence of consistent condom use, such behavior carries substantial STI risks (11,12). According to our findings, inconsistent condom use remains the dominant pattern of condom use among young Croatian adults. Adding to concerns, reports of consistent condom use may include instances of failed or flawed condom use, which were shown to be rather frequent (31). As expected, we noted a higher proportion of condom use at first than at most recent sexual intercourse, which is likely a consequence of relationship consolidation. The observed increase in the proportion of condom use at first intercourse is encouraging. Condom use at most recent intercourse appears, however, to be comparatively low. As opposed to this, recent studies among young people in Italy (29), Spain (32), and Canada (33) reported the prevalence of condom use at most recent intercourse to be higher than 70%.

In the context of contemporary sexuality, characterized by ubiquitous pre-marital sexual activity and sequential sexual relationships, a regular use of condoms remains one of the central protective behaviors. In multivariate analyses, three indicators were found to be robust (stable across time) and gender non-specific predictors of consistent condom use: younger age, positive attitudes toward condom use, and condom use at sexual debut.

Corroborating earlier findings (13,15,17), condom use at first sexual intercourse was found to be the strongest predictor of consistent condom use. Coupled with the recent evidence that consistent condom use may be the result of habit-formation (34), the finding underscores the importance of timing of sex education. To encourage consistent condom use, comprehensive school-based sex education would need to be introduced before the onset of coupled sexual activity. Focusing on communication and behavioral skills – including fostering condom use self-efficacy – sex education could promote responsible sexual behavior through delaying the first coital activity (to reduce the risks related to early sexual initiation) and assisting the process of condom use habit formation.

HIV incidence and prevalence remain comparably low in Croatia, with less than 0.1% infections and, on average, 10-15 new HIV infections per million a year in the last 10 years (35). This is probably the main reason for relatively little media attention that the epidemic receives. However, the existing data on the prevalence of STIs among young adults, although limited in scope, are at odds with the current inactivity in the area of HIV and STI prevention among the general population of young adults. Further delays of the introduction of systematic prevention of sexual risks in Croatia through school-based sexuality education programs may have serious epidemiological consequences.

Several study limitations need to be addressed. Despite adherence to standard procedures that maximize anonymity and confidentiality, validity of the findings reported in this article is limited by self-reporting. Even though proportions of individuals who refused to participate in the study because of its subject were almost identical in the 2005 and 2010 waves, a larger total non-response in 2010 suggests selection biases that may have affected generalizability and comparability of the findings. However, some recent analyses indicate that non-response does not necessarily increase biases (23), while several Nordic studies suggest that non-response in studies that investigate sexual behavior in youth is fairly random (36). Therefore, the extent to which systematic bias was introduced by low participation rate in 2010 remains unclear. Finally, validity of some indicators may have been affected by recall bias. The fact that most participants were at the beginning of their sexual careers reduces the likelihood of such bias. To further minimize this problem, a number of measures had reasonably short timeframe (usually 12 months).

Fully aware that the five-year timeframe may not be long enough to detect possible shifts in attitudinal and behavioral patterns, we firmly believe that continuous monitoring of the dynamics of HIV relevant knowledge, attitudes, and behaviors is a public health imperative. Systematic study of sexual risk taking, particularly among young people who are the most vulnerable segment of the general population, is crucial not only for tracing epidemiological trends but also for informing and guiding future prevention and intervention programs in the country. The present study is hopefully one of the first steps in this direction.

## References

[1] Christie D, Viner R (2005). Adolescent development.. BMJ.

[2] Panchaud C, Singh S, Feivelson D, Darroch JE (2000). Sexually transmitted diseases among adolescents in developed countries.. Fam Plann Perspect.

[3] Štulhofer A (1999). Terra Incognita? Adolescent sexuality and sexual risk-taking. Društvena Istraživanja..

[4] Impett EA, Tolman DL (2006). Late adolescent girls' sexual experiences and sexual satisfaction.. J Adolesc Res.

[5] Santelli JS, Morrow B, Anderson JE, Lindberg LD (2006). Contraceptive use and pregnancy risk among U.S. high school students, 1991-2003.. Perspect Sex Reprod Health.

[6] Braithwaite K, Thomas VG (2001). HIV/AIDS knowledge, attitudes, and risk-behaviors among African-American and Caribbean college women.. Int J Adv Couns.

[7] Štulhofer A, Jureša V, Mamula M (2000). Problematic pleasures: Sexual risk-taking in late adolescence. Društvena Istraživanja..

[8] Denissenko M, Dalla Zuanna G, Guerra D (1999). Sexual behavior and attitudes of students in the Moscow State University.. Eur J Popul.

[9] UNAIDS. Report on the global AIDS epidemic. Geneve (Switzerland): UNAIDS; 2008.

[10] Weinstock H, Berman S, Cates W (2004). Sexually transmitted diseases among American youth: incidence and prevalence estimates, 2000.. Perspect Sex Reprod Health.

[11] Marijan T, Vranes J, Mlinarić-Dzepina A, Leskovar V, Knezević J, Kvaternik M (2007). Genital human papillomavirus infection in women from the Zagreb region.. Coll Antropol.

[12] Milutin-Gasperov N, Sabol I, Halec G, Matovina M, Grce M (2007). Retrospective study of the prevalence of high-risk human papillomaviruses among Croatian women.. Coll Antropol.

[13] Stulhofer A, Graham C, Bozicevic I, Kufrin K, Ajdukovic D (2009). An assessment of HIV/STI vulnerability and related sexual risk-taking in a nationally representative sample of young Croatian adults.. Arch Sex Behav.

[14] Hirsl-Hecej V, Stulhofer A (2006). Condom use and its consistency among metropolitan high school students in Croatia, 1997-2001: has anything changed?. Coll Antropol.

[15] Štulhofer A, Ajduković D, Božičević I, Kufrin K. AIDS and youth – Croatia 2005 [in Croatian]. Zagreb: Ministarstvo zdravstva i socijalne skrbi, Hrvatski zavod za javno zdravstvo; 2006.

[16] Štulhofer A, Anterić G, Šlosar S (2004). Sexual permissiveness, egalitarianism, and responsible behavior: Longitudinal research of sexuality in late adolescence, 1998-2003. Revija za Sociologiju.

[17] Hirsl-Hecej V, Stulhofer A (2001). Urban adolescents and sexual risk taking.. Coll Antropol.

[18] Landripet I, Šević S, Car D, Baćak V, Mamula M, Štulhofer A (2010). Changing sexuality? Results from repeated cross-sectional studies of the University of Zagreb first-year students, 1998-2008. Društvena Istraživanja..

[19] Bijelić N (2008). Sex education in Croatia: tensions between secular and religious discourses.. Eur J Womens Stud.

[20] Lučin A, Dukić S. The state gave up on sex education [in Croatian]. Available from: http://www.slobodnadalmacija.hr/Hrvatska/tabid/66/articleType/ArticleView/articleId/ 34909/Default.aspx Accessed: July 5, 2011.

[21] Kish L (1949). A procedure for objective respondent selection within the household.. J Am Stat Assoc.

[22] Bagić D (2004). The influence of refusals on the validity of the pre-election telephone survey: The case of the 2003 parliamentary elections. Društvena Istraživanja..

[23] Šoša J, Milas G (2008). Slammed Door: Empirical Analysis of Survey Refusal Reasons. Društvena Istraživanja..

[24] UNAIDS. Monitoring the Declaration of Commitment on HIV/AIDS: Guidelines on construction of core indicators – 2008 reporting. Geneve (Switzerland): UNAIDS; 2007.

[25] Štulhofer A, Dokmanović D, Ajduković I, Božičević I, Kufrin K (2005). Youth sexuality in Croatia: symbolic and behavioral changes, 1972-2005. Pedagogijska Istraživanja..

[26] Ajduković D, Ajduković M, Prišlin R. AIDS and youth [in Croatian]. Zagreb: Medicinska naklada; 1991.

[27] Wellings K, Collumbien M, Slaymaker E, Singh S, Hodges Z, Patel D (2006). Sexual behaviour in context: a global perspective.. Lancet.

[28] Gelbal S, Duyan V, Ozturk AB (2008). Gender differences in sexual information sources, sexual attitudes, and behaviors of university students in Turkey.. Soc Behav Pers.

[29] Trani F, Gnisci F, Nobile CG, Angelillo IF (2005). Adolescents and sexually transmitted infections: knowledge and behaviour in Italy.. J Paediatr Child Health.

[30] Stock C, Guillen-Grima F, Prufer-Kramer L, Serrano-Monzo I, Marin-Fernandez B, Aguinaga-Ontoso I (2001). Sexual behavior and the prevalence of Chlamydia trachomatis infection in asymptomatic students in Germany and Spain.. Eur J Epidemiol.

[31] Baćak V Štulhofer A Condom use errors and problems in a national sample of young Croatian adults. Arch Sex BehavForthcoming2011 10.1007/s10508-011-9838-x 21882054

[32] Martin TC (2005). Contraceptive use patterns among Spanish single youth.. Eur J Contracept Reprod Health Care.

[33] Rotermann M (2008). Trends in teen sexual behavior and condom use.. Health Rep.

[34] Stulhofer A, Bacak V, Ajdukovic D, Graham C (2010). Understanding the association between condom use at first and most recent sexual intercourse: An assessment of normative, calculative, and habitual explanations.. Soc Sci Med.

[35] Croatian National Institute for Public Health. HIV/AIDS Epidemiological Situation in Croatia [in Croatian]. Available from: http://www.hzjz.hr/epidemiologija/hiv.htm Accessed: July 5, 2011.

[36] Traen B Štulhofer A Landripet I Young and sexual in Norway and Croatia: Revisiting the Scandinavian vs. Mediterranean gendered pattern of sexual initiation. Int J Sex HealthForthcoming2011

